# Functional expression and intracellular signaling of UTP-sensitive P2Y receptors in theca-interstitial cells

**DOI:** 10.1186/1477-7827-8-88

**Published:** 2010-07-14

**Authors:** Francisco G Vázquez-Cuevas, Erika P Zárate-Díaz, Edith Garay, Rogelio O Arellano

**Affiliations:** 1 Departamento de Neurobiología Celular y Molecular, Instituto de Neurobiología, Universidad Nacional Autónoma de México. Boulevard Juriquilla 3001, Juriquilla Querétaro, CP 76230, México

## Abstract

**Background:**

Purinergic receptors are expressed in the ovary of different species; their physiological roles remain to be elucidated. UTP-sensitive P2Y receptor activity may regulate cell proliferation. The aim of the present work was to study the functional expression of these receptors in theca/interstitial cells (TIC).

**Methods:**

TIC were isolated by centrifugation in a Percoll gradient. P2Y receptors and cellular markers in TIC were detected by RT-PCR and Western blot. Intracellular calcium mobilization induced by purinergic drugs was evaluated by fluorescence microscopy, phosphorylation of MAPK p44/p42 and of cAMP response element binding protein (CREB) was determined by Western blot and proliferation was quantified by [3H]-thymidine incorporation into DNA.

**Results:**

RT-PCR showed expression of p2y2r and p2y6r transcripts, expression of the corresponding proteins was confirmed. UTP and UDP, agonists for P2Y2 and P2Y6 receptors, induced an intracellular calcium increase with a maximum of more than 400% and 200% of basal level, respectively. The response elicited by UTP had an EC50 of 3.5 +/- 1.01 μM, while that for UDP was 3.24 +/- 0.82 μM. To explore components of the pathway activated by these receptors, we evaluated the phosphorylation induced by UTP or UDP of MAPK p44 and p42. It was found that UTP increased MAPK phosphorylation by up to 550% with an EC50 of 3.34 +/- 0.92 and 1.41 +/- 0.67 μM, for p44 and p42, respectively; these increases were blocked by suramin. UDP also induced p44/p42 phosphorylation, but at high concentrations. Phosphorylation of p44/p42 was dependent on PKC and intracellular calcium. To explore possible roles of this pathway in cell physiology, cell proliferation and hCG-induced CREB-phosphorylation assays were performed; results showed that agonists increased cell proliferation and prevented CREB-phosphorylation.

**Conclusion:**

Here, it is shown that UTP-sensitive P2Y receptors are expressed in cultured TIC and that these receptors had the ability to activate mitogenic signaling pathways and to promote cell proliferation, as well as to prevent CREB-phosphorylation by hCG. Regulation of TIC proliferation and steroidogenesis is relevant in ovarian pathophysiology since theca hyperplasia is involved in polycystic ovarian syndrome. Purinergic receptors described might represent an important new set of molecular therapeutic targets.

## Background

Theca cells form a multilayer cover that surrounds the follicle beginning in its early developmental stages. The main physiological roles recognized for theca cells are the initial steps in the steroidogenic process; specifically, these cells convert acetate or cholesterol to androgens [1], which are secreted into the intra-follicular medium and taken up by granulosa cells to serve as substrate for estrogen synthesis. In addition, theca cells could be an important signal integrator and regulator of aspects of follicular growth, because it represents the last follicular layer in contact with blood flow and receives chemical information from the peripheral nervous system [2].

Several studies in recent years indicate that the purinergic signaling system is functionally expressed in the ovary of several species [3] and represents another regulatory element in ovarian physiology; however, the physiological role of ATP in this context and its membrane receptors is unknown.

ATP is an important neurotransmitter in the peripheral nervous system [3], and nerve terminals from this system are potential sources for ATP release in the ovary. For example, the ovary is innervated by sympathetic terminals through the superior ovarian nerve and ovarian plexus [2,4]. It has been shown in other tissues that ATP is co-released with noradrenaline by sympathetic terminals and that it participates in several physiological events such as the induction and regulation of smooth muscle contraction [5] and the modulation of cardiac muscle excitation [6]. In addition, several cell types are able to release ATP in a basal manner and/or in response to different stimuli, such as mechanical stimulation, changes in pH, or hypotonic stress [7-9].

As a cellular messenger, ATP exerts its action through membrane receptors named P2, which are grouped into two subfamilies: P2X receptors that are cationic channels, and P2Y receptors that belong to the G protein-coupled receptor (GPCR) super family. In mammals, 8 subtypes of P2Y receptors have been described: 1, 2, 4, 6, and 11-14. Subtypes P2Y1, 2, 4, 6, and 11 are mainly coupled to Gα_q/11 _proteins, and they activate phospholipase C (PLC) and consequently diacylglycerol and phosphoinositide-Ca^2+ ^turnover; subtypes 12-14, on the other hand, are coupled to Gα_i/0 _proteins that signal primarily by inhibiting adenylyl cyclase [10].

P2Y2, P2Y4, and P2Y6 form a subgroup of receptors sensitive to uridine nucleotides [11]; P2Y2 and P2Y4 show selectivity for nucleoside triphosphates, while P2Y6 prefers mainly nucleoside diphosphates, specifically UDP [12]. Uridine P2Y-activated receptors are involved in a broad variety of physiological processes such as cell proliferation, smooth muscle contraction, transmitter release, and others [3,10]. In the ovary, expression of UTP-sensitive P2Y receptors has been described in granulosa luteal cells [13,14], in the cumulus cell-oocyte complex [15], and in *Xenopus *ovarian follicles [16,17].

Recently, it was demonstrated that functional P2X7 receptors are expressed in mammalian TIC and can induce apoptotic cell death [18]. In the same study, it was also observed that the application of UTP evoked intracellular [Ca^2+^]_i _changes, suggesting that multiple P2 receptor subtypes are expressed in theca cells. Here, we studied this response in order to elucidate in more detail the molecular elements involved and the physiological implications of their activation. We found that uridine-sensitive P2Y2 and P2Y6 receptors are expressed in the TIC membrane and that P2Y activation promoted three important responses in these cells: 1) elicited Ca^2+ ^mobilization from intracellular reservoirs, increasing the concentration of this important second messenger in the cytoplasm; 2) increased cell proliferation through a mechanism dependent on the activation of protein kinase C (PKC) as well as MAPK p44 and p42, and; 3) down regulated hCG-dependent phosphorylation of CREB, an important element in steroidogenesis cascade control.

## Methods

### Materials

ATP, UTP, UDP, suramin, human chorionic gonadotropic hormone (hCG), porcine follicle stimulating hormone (FSH), and PPADS were purchased from Sigma Chemical Co. (St. Louis, MO), and staurosporin, wortmanin, and phorbol 12-myristate 13-acetate (PMA) were from Calbiochem (Gibbstown, NJ). DMEM-F12 medium, fetal bovine serum, antibiotic-antimycotic mix, and other cell culture reagents were from Gibco Invitrogen Co. (Grand Island, NY). Antibodies against mouse total or phosphorylated MAPK p44 and p42 and total or phosphorylated CREB were from Cell Signaling (Danvers, MA), and antibody against poly(ADP-ribose) polymerase-1 (PARP) was from Santa Cruz Biotechnology (Santa Cruz, CA). Oligonucleotides, reverse transcriptase, oligo dT, *taq *polymerase, and other molecular biology reagents were purchased from Invitrogen Co. (Carlsbad, CA), and Fluo4-AM was from Molecular Probes Invitrogen Co. (Eugene, OR). Automatic sequencing was done in the Molecular Biology Unit of the Instituto de Neurobiología, UNAM.

### Theca cell isolation and culture

Mouse theca/interstitial cells were purified by a discontinuous Percoll gradient [19,20]. Immature mice were used to avoid cultures enriched in luteal cells. Thus, intact 4- to 5-week-old mice of the strain C57 were sacrificed by cervical dislocation, a procedure approved by the ethical committee of Instituto de Neurobiología-UNAM. The ovaries were dissected and incubated in collagenase (100 mg/ml) for 20 min, and the ovarian epithelium was removed by passing the complete ovary repeatedly in and out through the orifice of a Pasteur pipette. Most granulosa cells were then eliminated by puncturing the isolated, epithelium-free ovaries with a fine hypodermic needle. The ovary, free of epithelium and most granulosa, was cut into fine pieces that were then incubated in a mix of collagenase (4 mg/ml), DNase I (10 μg/ml), and BSA (10 mg/ml) for 30 min. The homogenate was fractioned on a discontinuous gradient: bottom layer 44% Percoll; top layer Percoll of density 1.055 g/ml. The cells were centrifuged for 20 min at 400 × g, and TIC were collected from the interface by aspiration, then cultured in DMEM-F12 medium containing 10% fetal bovine serum supplemented with antibiotic-antimycotic at 37°C in a humidified atmosphere with 5% CO_2_. Cultures were maintained 48 h before using them in experiments, to allow proper recovery after the isolation procedure. With this method we usually obtained 1 × 10^6 ^cells per mouse; purity was assayed by RT-PCR and immunocytochemistry against CYP11A protein (> 96% of cells were positive). To exclude contamination with granulosa, the expression of the FSH receptor transcript and the responsiveness of CREB phosphorylation to hCG or FSH were assayed.

### Reverse Transcription Polymerase Chain Reaction

Total RNA of TIC cultures or from the indicated organ was purified using the guanidine isothiocyanate method [21]. First strand cDNA was synthesized using 2 μg of DNase-treated RNA as template, 1 mg of oligo(dT), random hexamers, and reverse transcriptase.

The cDNA was used as template in a polymerase chain reaction to amplify cDNA fragments for *β-actin*, *p2y2r*, *p2y4r*, and *p2y6r *transcripts, and for *cyp11A, cyp17A, star*, and *fshr *as positive and negative theca cell markers, respectively. All the PCR programs started at 96°C for 3 min and finished at 72°C for 1 min. The amplification cycles consisted in 40 s at 96°C, 40 s at the specific annealing temperature for each primer set, and 40 s at 72°C.

The sequences of the oligonucleotides, the annealing temperatures, and the number of PCR cycles used were as follows: *p2y2r*, forward GGACGAACTGGGATACAAGTGT, reverse GTGGACTCTGTCCGTCTTGAGT, annealing temperature 55°C, 30 cycles; *p2y4r*, forward GGGACTAACTGCAGGCAGAG, reverse GATACACATCAGGCCCGTCT, annealing temperature 60°C, 40 cycles; *p2y6r*, forward TTTCAAGCGACTGCTGCTAA, reverse TGGCATAGAAGAGGAAGCGT, annealing temperature 55°C, 30 cycles; *cyp11A*, forward GCTGGAAGGTGTAGCTCAGG, reverse CACTGGTGTGGAACATCTGG, annealing temperature 55°C, 30 cycles; *cyp17A*, forward TGGTCGGCCCCAGATGGTGA, reverse ATCTCGGGACTCCCCGTCGT, annealing temperature 56°C, 30 cycles; *star*, forward AACCAGGAAGGCTGGAAGAAG, reverse AGCAGCCAAGTGAGTTTAGTC, annealing temperature 55°C, 30 cycles; *fshr*, forward TGGATGTCATCACTGGCTGT, reverse CAAATCTCAGTTCAATGGCG, annealing temperature 58°C, 30 cycles; and *β-actin*, forward GGGTCAGAAGGATTCCTATG, reverse GGTCTCAAACATGATCTGGG, annealing temperature 55°C, 25 cycles.

The amplified products were gel-isolated, phenol-chloroform purified, and subcloned into the pCR4-TOPO vector (Molecular Probes Invitrogen Co.). Their nucleotide sequences were confirmed by automatic sequencing.

### Fluorescence microscopy

Mouse ovarian TIC were grown on 12-mm diameter cover slides. Semi-confluent cultures were loaded for 15 min with 5 mM fluo-4/AM and 0.1% pluronic acid in Krebs solution (in mM: 150 NaCl, 1 KCl, 1 MgCl_2_, 1.5 CaCl_2_, 4 glucose, 10 HEPES, and 0.05% BSA, pH 7.4). The cells were washed with Krebs solution for 10 min to eliminate excess dye and then placed in a constant-flow recording chamber that allowed them to be visualized with an inverted fluorescence microscope (Olympus IX70, Melville, NY). Drugs were applied by superfusion and responses were recorded with an Evolution QEi camera (Media Cybernetics, Bethesda, MD). Sequences of images were analyzed using the Image-Pro Plus software (Media cybernetics, Bethesda, MD) and Imagenes software, a program developed specifically for this analysis (Dr. Ivan Terol, CIDETEQ, México). In the Ca^2+^-free Krebs solution, CaCl_2 _was replaced by 3 mM MgCl_2_.

### Western blot

For MAPK p42 and p44 or CREB phosphorylation experiments, cultured TIC (5 × 10^5^) were harvested 24 h before the experiment to reduce serum-dependent kinase activity. After that, cells were stimulated with the indicated drugs, scraped in Laemmli buffer, and boiled for 5 min. For electrophoresis, samples were fractionated in a 10% SDS-polyacrylamide gel and transferred to a nitrocellulose membrane (BioRad, Hercules, CA). Membranes were blocked for 1 h at room temperature in 150 mM NaCl, 20 mM Tris, pH 7.4, and 0.1% Tween 20 (TBS-T) containing 5% nonfat dry milk and then incubated overnight at 4°C with the appropriate rabbit primary antibody (1:1000) directed against the phosphorylated form of MAPK p44 and p42 or CREB (Cell signaling, Danvers, MA). After washing with TBS-T, membranes were incubated 1 h at 37°C with HRP-conjugated goat anti-rabbit antibody (Zymed, Invitrogen Co., Grand Island, NY) in TBS-T. The immunoreactive proteins were detected by chemiluminescence, and images were analyzed by pixel density with ImageJ Software (NIH, USA); the results were expressed in terms of optic density normalized against the basal condition, a parameter that is proportional to the change in protein phosphorylation. To analyze total p44/p42 or other load controls, such as PARP, the same membranes were incubated for 30 min in striping solution (50 mM, Tris pH 6.8, 100 mM β-mercaptoethanol, and 2% SDS) at 55°C, washed twice with TBS-T, and then reprobed with a primary antibody against the indicated protein.

### Immunoprecipitation

TIC (1 × 10^6^) were scraped in ice-cold TNTE buffer (Tris-HCl pH 7.4,150 mM NaCl, 50 mM and 0.5 mM EDTA) containing 5% Triton X-100 and a protease inhibitor cocktail (Roche Co., Basel, Switzerland); the lysate was centrifuged for 10 min at 14,000 rpm at 4°C, and the soluble fraction was incubated overnight with 3 μl of anti-P2Y6 antibody (Alomone, Jerusalem, Israel). After that, 50 μl of protein G agarose was added to the lysate and incubated for 1 h at room temperature; the agarose beads were washed 3 times with TNTE containing 1% Triton X-100 and protease inhibitors, resuspended in Laemmli buffer, boiled for 5 min, and analyzed by Western blot.

### Proliferation assay

Cell proliferation was analyzed using [^3^H]-thymidine incorporation. For this, cells (10^4^) were cultured in 48-well plates and after 48 h of culture, they were harvested and incubated for 24 h in serum-free DMEM-F12 media; then the culture medium was changed to DMEM-F12 with 0.1% fetal bovine serum containing the experimental treatment (UTP, UDP, or ATP at the indicated concentration). Then cultures were incubated for another 48 h, with the addition of 1 μ Ci/well of [^3^H]-thymidine after the first 24 h. At the end of the incubation, each well was washed 3 times with 5% trichloroacetic acid, and then the cells were lysed by addition of 250 μl of boiling 250 mM NaOH, incubated 5 min, and transferred to vials containing 5 ml of scintillation liquid. Samples were counted in a scintillation counter.

### Statistical analysis

All data are expressed as mean ± S.E.M. Statistical analysis was performed using GraphPad Prism (La Jolla, CA) software. The means of two groups were compared using a Student's t-test. ANOVA was used to compare several groups, and differences were considered to be significant at p < 0.05.

## Results

### Theca cell identity and expression of P2Y2, P2Y4, and P2Y6 receptors

**Figure 1:**
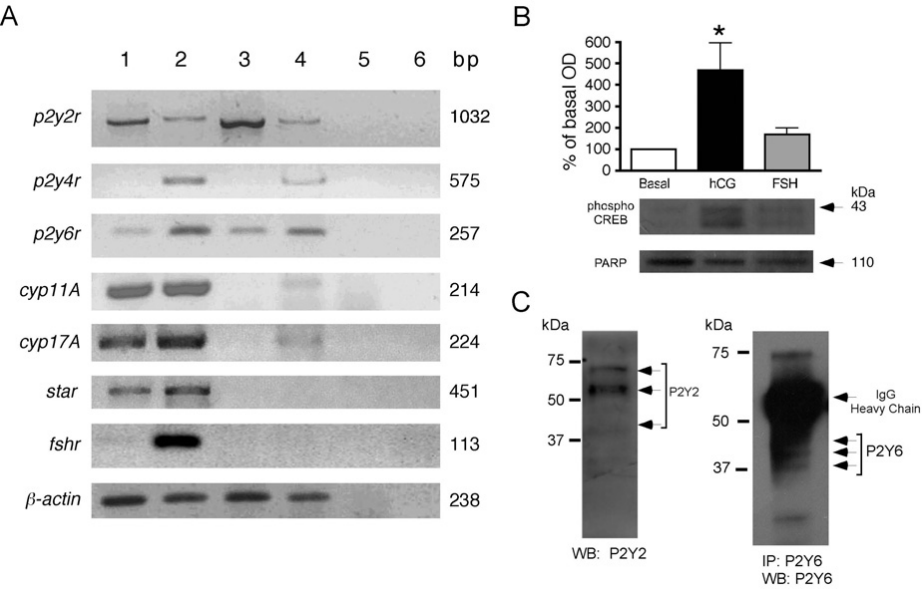
***p2y2r, p2y4r*, and *p2y6r *expression in mouse theca cells**. (A) RNA from cultured theca cells (1), whole ovary (2), heart (3), and brain (4) was reverse transcribed and amplified by PCR for *p2Y2, p2Y4, p2Y6, cyp11A, cyp 17A, star, fshr, and β-actin *using specific oligonucleotides to detect transcript expression. Controls in lanes (5) and (6) correspond to a PCR reaction of a theca cell RNA sample without reverse transcriptase and to the reaction mix without a cDNA template, respectively. Amplicon length is indicated in base pairs (bp). Each amplification assay was repeated in 3-5 independent cultures. (B) CREB phosphorylation evaluated by Western blot from TIC cultures in basal conditions or stimulated for 10 min by either 2 IU hCG or 1 ng/ml FSH. The 43-kDa band was analyzed by densitometry, and the result (mean ± S.E.M) of three determinations from independent cultures was plotted (*p < 0.01). PARP protein was used as gel loading control. (C) P2Y2 and P2Y6 receptors detected by Western blot (WB), from freshly isolated TIC homogenates directly (P2Y2) or after immunoprecipitation (IP) (P2Y6).

TIC were isolated, and their identity was confirmed by RT-PCR amplification of *cyp11A, cyp17A, and star *transcripts as specific markers for theca cells, and of FSH receptor (*fshr*) transcripts as indicator of a possible contamination with granulosa cells; the *β*-actin transcript was used as a control housekeeping gene (Figure 1A). The results showed that TIC cultures were positive for *cyp11A, cyp17A, and star *expression, but they did not express the FSH receptor, demonstrating that the isolated cells were mainly of the thecal/interstitial type and were essentially free of granulosa cells.

This conclusion was strengthened with data obtained in functional experiments. For this, TIC cells were stimulated by 2 IU hCG or 1 ng/ml FSH, and CREB phosphorylation was evaluated. It is well established that gonadotropin receptors exert their actions by coupling to G proteins, increasing cAMP synthesis that, in consequence, promotes CREB-phosphorylation [22,23]. It was found that in TIC cultures, CREB protein-phosphorylation (43-kDa band) was increased 6-fold by hCG stimulation, whereas FSH did not induce any change (Figure 1B), thus supporting the idea that these cultures contained mainly theca/interstitial cells.

To study the expression of *p2y2r, p2y4r*, and *p2y6r *transcripts, RNA from TIC was reverse transcribed, and then PCR was carried out with specific oligonucleotides for each receptor subtype. RNA samples from ovary, brain, and heart were also analyzed as controls. As shown in Figure 1A, a *p2y2r *fragment of 1032 bp and a *p2y6r *fragment of 257 bp were amplified from the cDNA of all tissues tested. However, the *p2y4r *fragment of 575 bp was only amplified from the whole ovary and brain cDNA. In all the assays, control amplifications without RT or without cDNA template did not produce any PCR product (Figure 1A). The amplified fragments were cloned into the pCR4-TOPO vector, sequenced, and analyzed in BLAST, and the fragments were identical to the reported sequences from mouse (NM008773 for *p2y2r*, NM020621 for *p2y4r*, and NM183168 for *p2y6r*).

These RT-PCR results indicated that TIC might express P2Y2 and P2Y6 receptors. In order to detect the protein, Western blot was performed from homogenates to detect P2Y2; to detect P2Y6 receptor it was necessary to perform immunoprecipitation followed by Western blot, which suggested a low expression level of this receptor (Figure 1C). P2Y2 was detected as a band of 58 kDa, a major band near 70 kDa, and a fainter band of 45 kDa. P2Y6 was detected as three bands with molecular weights of approximately 45, 40, and 37 kDa. In the latter case, the IgG heavy chain interfered with the immunoreactive bands corresponding to the receptor. However, all bands observed match the molecular weights reported previously for both receptor types [e.g., [24,25]].

### UTP- and UDP-induced increase of intracellular Ca^2+ ^concentration in TIC

**Figure 2:**
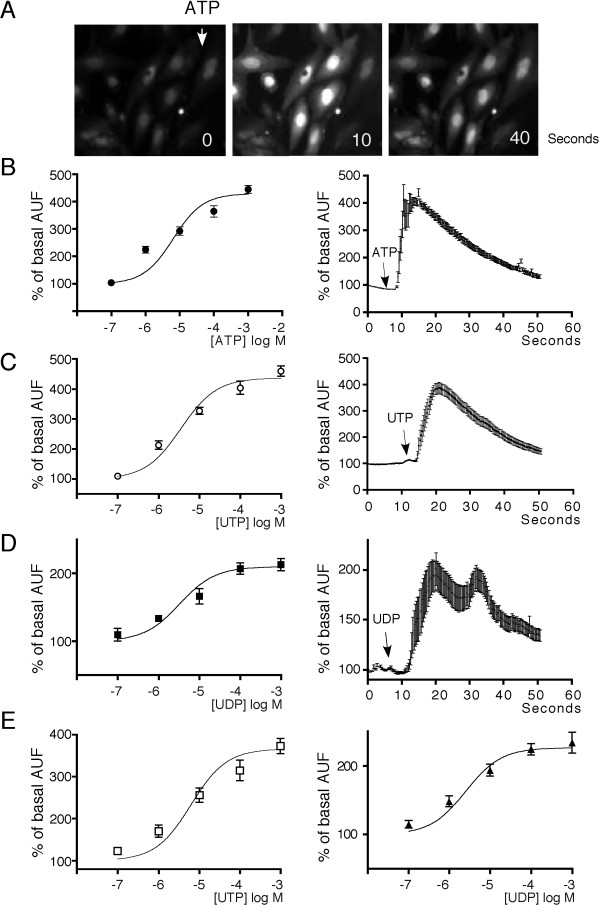
**Dose-response (D-R) relationships and time course of intracellular Ca^2+ ^responses generated by ATP, UTP, or UDP in TIC cells**. (A) Images show a typical fluorescence increase of theca cells loaded with Fluo4-AM dye upon stimulation with 100 μM ATP at 0, 10, or 40 s. (B) D-R relationship (left panel) for the intracellular Ca^2+ ^increase elicited by ATP, expressed as a percent of the basal level of arbitrary units of fluorescence (AUF), and time course of the response elicited by 1 mM ATP (right panel). (C) and (D) show experiments similar to those in (B) but applying UTP or UDP, respectively, instead of ATP. (E) D-R curves of the theca cell response to UTP (left) or UDP (right) in Ca^2+^-free Krebs. The stimulus was applied at the time indicated by an arrow and was maintained to the end of the recording. Data points represent the mean ± S.E.M. from 25 to 40 cells in each of 3 independent experiments.

Functional responses of P2Y receptors were studied by applying ATP, UTP, or UDP to TIC and monitoring the changes in intracellular calcium concentration ([Ca^2+^]_i_) using fluorescence microscopy of Fluo-4-AM loaded-cells. In all cases, 25 to 40 cells from 3 independent cultures (each from 2 donors) were analyzed (Figure 2).

Figure 2A shows a typical response elicited by 100 μM ATP. At the highest concentration tested (1 mM), ATP elicited a [Ca^2+^]_i _increase of 458 ± 18% compared with the basal level (Figure 2B, left panel); this increase was monotonic (Figure 2B, right panel), dose-dependent, and had an EC_50 _of 6.5 ± 1.0 μM. In cells from the same cultures, UTP also induced a dose-dependent response with an EC_50 _of 3.5 ± 1 μM and a maximal increase of 437 ± 12% (Figure 2C, left panel). As illustrated in the right panel, the increase generated by UTP had a similar time-course to that elicited by ATP.

Three types of P2Y receptors sensitive to UTP have been described: P2Y2, P2Y4, and P2Y6 receptors. UDP is a more potent agonist for P2Y6 receptors than UTP or ATP; thus, in order to detect a possible participation of P2Y6, TIC were tested with UDP. This nucleotide elicited responses with an EC_50 _3.2 ± 0.8 μM; however, the maximal response reached was only 210 ± 5.4% (Figure 2D, left panel). Furthermore, the [Ca^2+^]_i _increase in response to UDP consistently showed an oscillating time-course (Figure 2D, right panel), different from that observed with ATP or UTP.

In the absence of extracellular Ca^2+ ^(Ca^2+^-free solution), responses to either UTP or UDP were not abolished (Figure 2E). Nevertheless, maximal responses generated by UTP averaged 366 ± 13%, significantly less (P < 0.01) than those observed in normal Krebs solution (437 ± 12%). The EC_50 _obtained for UTP in Ca^2+^-free solution was 6.2 ± 0.9 μM and was not significantly different from that obtained in normal Krebs. For UDP, similar findings were observed: the maximal response reached 230 ± 15% and had an EC_50 _of 4.9 ± 0.6 μM; neither parameter differed significantly from that in normal Krebs.

This suggested that extracellular Ca^2+ ^was not the major source of the [Ca^2+^]_i _increase produced in TIC by UTP or UDP; more probably, this increase came from intracellular reservoirs via IP_3 _synthesis, as shown in other cell systems.

### UTP-induced activation of p44 and p42 MAPK

**Figure 3:**
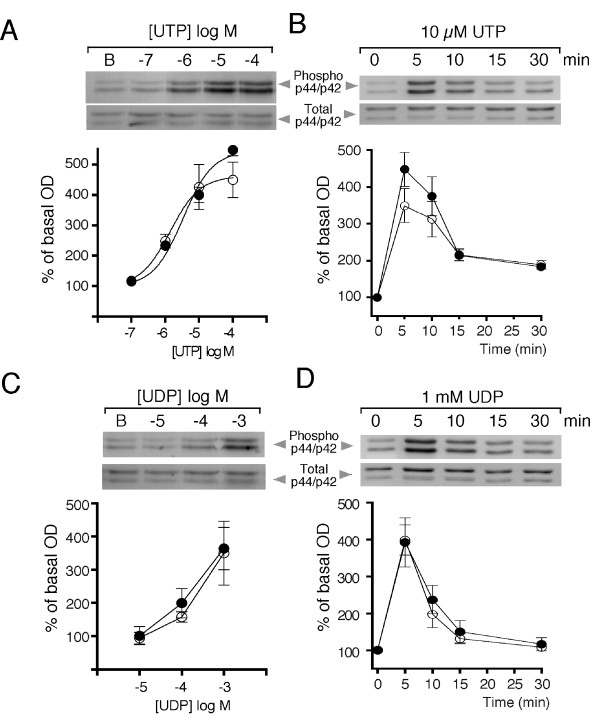
**D-R relationships and time course for the phosphorylation response elicited by UTP or UDP on p44 and p42 MAPK**. **(**A) Phosphorylated isoforms of p44 (black dots) or p42 (white dots) were immunodetected (Western blot) in samples of theca cells that were stimulated with various UTP concentrations. The band intensities (OD) are expressed as a percent of the basal level, and the values were used to plot the D-R relationship. (B) Time course of the p44 and p42 phosphorylation process elicited by 10 μM UTP. (C) D-R for phosphorylation elicited by UDP in experiments similar to those in (A). (D) Time course of p44 and p42 phosphorylation elicited by 1 mM UDP. Data points are the mean ± S.E.M. of three independent experiments.

In order to study the signaling pathway involved in the UTP and UDP activation of P2Y receptors in TIC, phosphorylation of the p44 and p42 MAPK proteins was evaluated (in at least 3 experiments, each in duplicate, carried out on independent cultures derived from pooled ovaries of 2-3 mice). For these experiments, UTP was used as a specific agonist of the P2Y receptor subtypes studied. It was observed that UTP induced MAPK phosphorylation in a dose-dependent manner with an EC_50 _of 3.3 ± 0.9 and 1.4 ± 0.7 μM for p44 and p42, respectively; maximal increases of 541 ± 25.6% and 461 ± 34.8%, respectively, were observed by applying 100 μM UTP (Figure 3A). The time course of this effect was studied by applying 10 μM UTP and measuring p44 and p42 MAPK phosphorylation at different times. The results indicated that maximal phosphorylation occurred at 5 min of stimulation, and then it decreased slowly, returning to near-basal levels about 30 min after UTP addition (Figure 3B).

**Figure 4:**
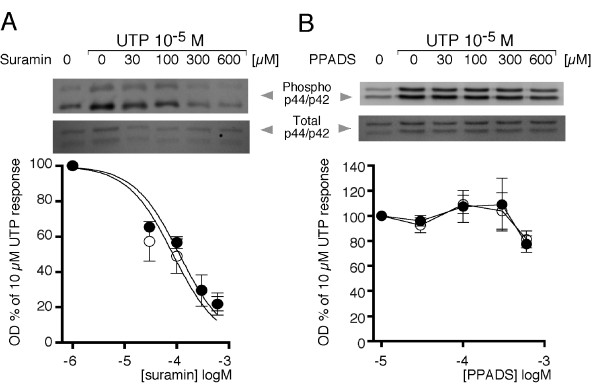
**p44 and p42 MAPK phosphorylation induced by UTP is blocked by suramin but not by PPADS**. Cultures of mouse theca cells were incubated for 30 min with suramin (A) or PPADS (B) at the indicated concentrations; subsequently, 10 μM UTP was applied for 5 min, and p44 (black dots) and p42 (white dots) MAPK phosphorylation was detected by Western blot. Data points are the mean ± S.E.M. of three independent experiments.

Because it has been shown consistently that UDP acts more potently on P2Y6 receptors [11,12], its ability to promote p44 and p42 MAPK phosphorylation was tested. In experiments similar to those presented above for UTP, 100 μM UDP was less potent and induced only modest responses of 199 ± 43% and 158 ± 15% for p44 and p42, respectively, compared to the basal level; the effect increased to 364 ± 63% and 349 ± 95%, respectively, with 1 mM UDP (Figure 3C). The time course of p44/p42 phosphorylation induced by 1 mM UDP was similar to that elicited by 100 μM UTP (Figure 3D). In addition, the p44 and p42 MAPK phosphorylation induced by 10 μM UTP was antagonized by suramin with an IC_50 _of 84.3 ± 10.2 μM (Figure 4A); suramin is a potent antagonist of P2Y2 receptors but is only a weak antagonist of P2Y6 [11]. Conversely, PPADS up to 600 μM, a drug that antagonizes mainly the P2Y6 receptor [11], had no effect on UTP-induced MAPK phosphorylation (Figure 4B). These results suggested that P2Y6 is not a major participant in the phosphorylation of MAPK; in consequence, the following experiments focused on defining the role of the P2Y2 receptor in the purinergic response.

### UTP-induced p44 and p42 MAPK phosphorylation is dependent on PKC activation

**Figure 5:**
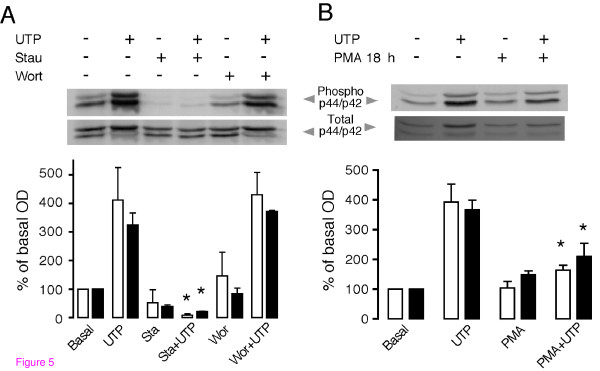
**Effect of protein kinase inhibitors on p44 and p42 MAPK phosphorylation induced by UTP**. (A) Western blot detection of phosphorylated p44 (white columns) and p42 (black columns) MAPK in the presence of 100 nM wortmanin or 250 nM staurosporine in theca cells stimulated or not with 10 μM UTP (5 min). In (B), down regulation of PKC was induced by an overnight (18 h) cell incubation with 1 μM PMA, and then phosphorylation of p44 and p42 MAPK was assayed for cells treated or not with 10 μM UTP (5 min). Data points represent the average optical density (± S.E.M.) of three independent experiments expressed as a percent of the basal level (*p < 0.05 vs. UTP, one-way ANOVA and Bonferroni's test).

UTP-dependent p44/p42 MAPK phosphorylation might be elicited via either of two main mechanisms: 1) transactivation of EGF receptors as has been demonstrated, for example, in salivary gland cells [26] or 2) by activation of kinases through its canonical pathway. However, EGF transactivation requires long-term (over 1 h) stimulation with the agonist, and activation of p44/p42 MAPK in TIC was generated even with short incubations; thus, it was decided to analyze first the role of down stream kinases. Two of the main candidate protein kinases, PKC and PI3K [27,28], were blocked using specific pharmacological tools. PI3K activation was blocked by preincubation for 30 min with the selective inhibitor 100 nM wortmanin (Figure 5A) or with 1 μM LY294002 (not shown) before stimulating the cells with 10 μM UTP; under these conditions, neither inhibitor had any effect on p44 or p42 MAPK phosphorylation (n = 3 independent cultures).

To study the possible participation of PKC, TIC cultures were preincubated (30 min) with 250 nM staurosporine and then tested with 10 μM UTP. Staurosporine treatment blocked completely the UTP-stimulated p44 and p42 phosphorylation (Figure 5A), strongly suggesting that phosphorylation was dependent on PKC. To support this idea, experiments were carried out in which PKC activity was downregulated by long-term incubations with phorbol-12-myristate-13-acetate (PMA) [29]. Thus, TIC were pretreated for 18 h with 1 μM PMA, which did not affect the basal levels of phosphorylated p44 or p42 proteins; cells were then stimulated with 10 μM UTP. Under these conditions, PMA preincubation reduced p44 and p42 MAPK phosphorylation induced by UTP from a maximal response without PMA of 347 ± 58% and 299 ± 56% for p44 and p42, respectively, to 164 ± 16% and 210 ± 43% (p < 0.05) (Figure 5B). These results indicate that PKC is the main kinase responsible for the UTP-induced activation of the p44 and p42 proteins.

**Figure 6:**
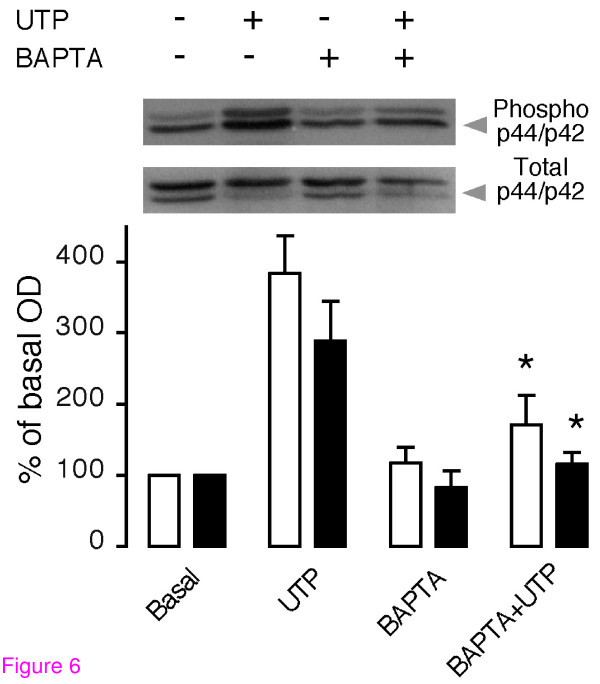
**Effect of intracellular BAPTA loading on p44 and p42 MAPK phosphorylation induced by UTP**. Cultures of mouse theca cells were preincubated for 90 min with 10 μM BAPTA-AM; cells were then stimulated for 5 min with 10 μM UTP and assayed for phosphorylation of MAPK p44 (white columns) and p42 (black columns). Amplitude bars in the plot represent the mean ± S.E.M. of three independent experiments (*p < 0.05 vs. UTP, one-way ANOVA and Bonferroni's test).

To test the role of intracellular Ca^2+ ^during p44 and p42 MAPK phosphorylation, cell cultures were preincubated (90 min) with 10 μM BAPTA-AM to load the cells intracellularly with this Ca^2+ ^chelator. Fluorescence microscopy confirmed that this treatment prevented the calcium increase induced by UTP. In BAPTA-loaded TIC, phosphorylation of MAPK elicited by UTP was strongly inhibited (Figure 6); thus, in control conditions, UTP elicited a phosphorylation increase of 384 ± 53 and 289 ± 55% for p44 and p42, respectively, while UTP-stimulated BAPTA-loaded cells showed significantly (p < 0.05) lower phosphorylation increases of only 171 ± 40 and 116 ± 16%, respectively. This result indicates that phosphorylation was a Ca^2+^-dependent process and provides evidence for PKC participation.

### Evidence that suggests a role for purinergic signaling in TIC physiology

**Figure 7:**
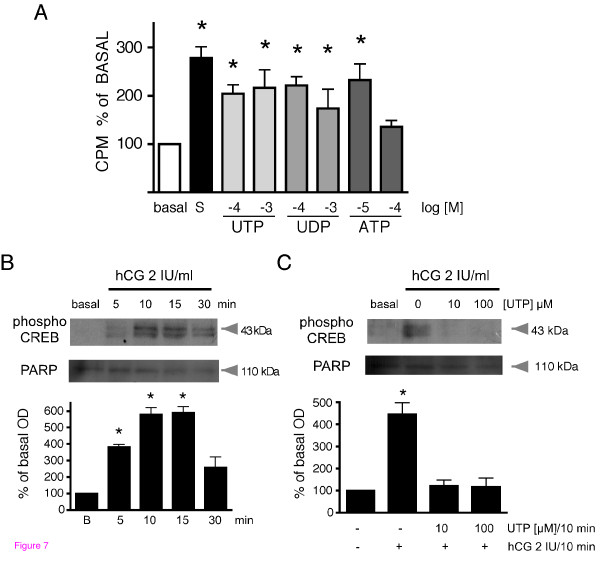
**Proliferative response and inhibition of hCG-induced CREB phosphorylation by P2Y agonists in mouse theca cells**. Theca cells were stimulated for 48 h with UTP, UDP, or ATP using the indicated concentrations (log M); controls corresponded to cells incubated in media supplemented with 0.1% serum (basal) or with 10% FBS (S). Cell proliferation was evaluated by [^3^H]-thymidine incorporation, data are the mean ± S.E.M. of 4 independent determinations. B) Time-course of CREB phosphorylation in TIC induced by 2 IU hCG; the 43-kDa band was quantified as in Figure 1B showing that maximal effect was reached in 10-15 min of incubation. C) CREB-phosphorylation elicited by 2 IU hCG in TIC incubated for 10 min in basal conditions and in the presence of 0, 10, or 100 μM UTP; the 43-kDa band was quantified as in B), data are from 3 independent experiments in triplicate. (*p < 0.05 vs. basal, one-way ANOVA and Bonferroni's test).

Cell proliferation is a consequence of purinergic stimulation in various cell systems [3]; here we asked whether or not P2Y stimulation of TIC induced their proliferation. For this, cell cultures were stimulated with different concentrations of UTP, ATP, or UDP; cell proliferation was estimated using [^3^H]-thymidine incorporation and compared with that elicited by 10% FBS (Figure 7 shows four independent determinations, each in triplicate, from cultures where the ovaries of 2-3 mice were pooled).

The results indicated that ATP, UTP, or UDP increased proliferation. Incubation with 10% FBS increased [^3^H]-thymidine incorporation to 277 ± 23% of the basal level, and a similar increase was induced by 10 μM ATP (232 ± 33%) but not by 100 μM ATP (135 ± 13%). Also, 100 μM UTP or UDP induced robust responses of 205 ± 18% and 221 ± 17%, respectively, while at 1 mM they generated increases of 216 ± 37% and 183 ± 23%, respectively. The results strongly suggested that P2Y receptors were able to activate a proliferative response in TIC.

The possibility of regulatory cross talk between P2Y2 and LH receptors was also examined. First, it was shown that TIC responded to a 2 IU hCG stimulus by increasing CREB phosphorylation, and this response reached a maximum in 10 min (figure 7B). This observation demonstrated that the LH receptor efficiently activated the cAMP pathway in TIC cultures (see figure 1B). It is well established that the cAMP/PKA/CREB pathway participates in the canonical signal transduction cascade for regulation of androgen biosynthesis through the LH receptor [22].

With this rationale, cultures of TIC were incubated in 0, 10, or 100 μM of UTP for 10 min, then 2 IU hCG were added for 10 min more, cell lysates were obtained, and CREB phosphorylation was evaluated by Western blot as an indicator of LH receptor activity. It was found that UTP, either 10 or 100 μM, completely blocked CREB phosphorylation (figure 7C), strongly suggesting that P2Y2 receptor stimulation inhibited the cAMP pathway activated by LH. This raises the possibility that the purinergic system participates in regulating the physiological actions promoted by LH, for example, the steroid hormones synthesis pathway.

## Discussion

It has been recognized that neurotransmitters might play distinct, regulatory roles in ovarian physiology; for example, it has been proposed that, in addition to the regulatory actions of gonadotropins, the activity of sympathetic fibers that innervate the ovary controls different aspects of ovarian function, such as steroidogenesis, folliculogenesis, and ovulation [e.g., [30,2,32]]; most of these studies have examined the role of the catecholaminergic system and specifically, of norepinephrine [30-32], but there is also a great deal of important evidence for participation of an ovarian cholinergic system [33,34]. Although knowledge about the purinergic system in the ovary is scarce, it is well established that ATP and norepinephrine are co-released at similar concentrations from sympathetic terminals in many cell systems [5,7], and release of ATP by the oocyte has already been documented in other species [8,16]. Thus, the study in the ovary of the molecular components expressed and cellular mechanisms activated by the purinergic system will be of importance to understand the possible role of ATP in ovarian physiology and pathology. Here, we present clear evidence of functional expression of UTP-sensitive P2Y receptors in TIC cultures, suggesting a role for these receptors in ovarian physiology.

RT-PCR and Western blot studies indicated that cultured TIC express P2Y2 and P2Y6 receptors. In functional experiments, UTP and UDP, specific agonists for P2Y2 and P2Y6, respectively, induced robust Ca^2+ ^signals in normal Krebs or in Ca^2+^-free solution, which indicated that the nucleotides promoted the response mainly through Ca^2+ ^release from intracellular reservoirs, in agreement with the canonical Gαq-PLC pathway for these receptors [10]. UTP or high concentrations of UDP (1 mM) also induced the phosphorylation of MAPK p44 and p42; at high concentrations, UDP acted principally on the P2Y2 receptor, since P2Y6 is stimulated by UDP in the low μM range [11]. Phosphorylation of MAPK was inhibited by suramin, a potent antagonist for P2Y2 and weak for P2Y6, but it was not affected by PPADS, which is inactive toward P2Y2 but able to antagonize P2Y6 activation [11]. Taken together, our data indicated a main role of the P2Y2 receptor in MAPK activation. There is ample evidence that these protein kinases are involved in the proliferative phenomenon activated by G protein-coupled receptors in various cell systems [e.g., [27,28]]; in addition, p44 and p42 MAPK activation dependent on P2Y2 or P2Y6 receptors has been described, e.g., in granulosa-luteal cells [14], glioma cells [35], and embryonic stem cells [36]. Staurosporin or long-term (18 h) incubation with PMA blocked UTP-induced p44 and p42 MAPK phosphorylation. In addition, p44 and p42 MAPK phosphorylation was blocked in BAPTA-loaded cells, strongly suggesting that a calcium-dependent PKC participates in this response.

Activation of MAPK p44 and p42 is directly related to induction of cell proliferation [37]. Our results demonstrated that UTP and UDP induced a robust proliferative response similar to that of 10% FBS used as positive control. ATP induced a proliferative response at 10 μM, but no effect was observed with higher concentrations. This supports the idea that P2Y2 is the main receptor involved in the response, but an ancillary participation of P2Y6 cannot yet be excluded. The regulation of theca cell proliferation is relevant during folliculogenesis [1], and it might be involved in pathological processes, such as the altered androgen-estrogen balance associated with polycystic ovary syndrome, a common disease characterized by uncontrolled theca cell proliferation [38]. In this context, purinergic signaling can activate a feedback mechanism by inducing a proliferative or an apoptotic response in TIC.

ATP actions to stimulate TIC proliferation through P2Y2 (and P2Y6) receptor activation should be taken into account, together with the effects described for other neurotransmitters that seem to regulate specific processes in the ovary. For example, previous evidence showed that human granulosa-luteal cells express M1 and M5 muscarinic receptors [34] as well as P2Y2 purinergic receptors [13,14]; stimulation of either system by acetylcholine or ATP can promote granulosa-luteal cell proliferation. Stimulation of β-adrenergic receptors also modulates steroidogenic activity and ovulation [31] and, given that neurotransmitters released from catecholaminergic terminals might include ATP, it would be of interest to know the effect of activating purinergic receptors in these processes.

The results also showed that P2Y2 receptor activation had an important effect on the LH signaling pathway. It has been shown before that LH induces CREB phosphorylation [22,39] and that expression of a dominant negative CREB variant was enough to block androgen biosynthesis in rat TIC cells [22]. We observed that preincubation with UTP (10 or 100 μM), completely blocked the hCG-induced CREB phosphorylation, which suggests that the purinergic system potently modulates LH-activated pathways, an action that might have important consequences in ovarian theca physiology.

Is well known that during folliculogenesis LH exerts regulatory actions beginning around the formation of early secondary follicles, which is concurrent with theca layer differentiation; from this stage throughout folliculogenesis up to ovulation, LH is the main regulator of theca layer development, because it controls the steroidogenesis process [40]. However, during this period, important phenomena such as follicular selection or dominance processes cannot be explained solely by LH action; paracrine and autocrine follicular molecules seem to be essential for the final outcome [41]. It is possible that P2Y2 activation represents one of the mechanisms by which LH regulates the cohort of follicles that will or will not become dominant. Thus, the process of purinergic regulation demonstrated here might be involved in maintaining the proper balance between the rate of cell division and death in the ovary, and in essential physiological actions such as steroidogenesis, functioning as a local, fine-tuning modulator to complement the systemic control exerted by hormones and nervous system afferents. Hence, purinergic regulation is a potential therapeutic target in ovarian pathologies where proliferation or the steroidogenesis processes are affected.

Specifically in regulating the balance between theca proliferation and death, our data suggest that activation of the purinergic system by ATP could have dual effects on theca cell physiology; i.e., depending on the concentration, ATP might induce: 1) apoptotic cell death through P2X7 receptors (18) and 2) cell proliferation through P2Y2/P2Y6 receptors, as shown here. This is similar to what has been demonstrated in other systems in which the cells seem to co-express multiple purinergic receptor subtypes, leading to activation of multiple signaling pathways. For example, macrophages express a variety of P2X and P2Y purinergic receptors, and their activation modulates diverse physiological process such as apoptosis, activation of cell proliferation pathways, or activation of the inflammatory response machinery [42,43]. The final physiological outcome of the effect exerted by ATP in a given process will be determined by several factors including, for example, the purinergic receptor affinities, source and availability of ATP, ecto-ATPase activity, and also cross-talk between different G protein-coupled receptor types or subunits of receptor channels [see e.g., [17]]. In this context, it is important to mention that high concentrations of ATP (0.1 - 1 mM), but not of UTP, were consistently unable to increase cell proliferation, which might be a result of P2X7 receptor activation that can induce apoptotic cell death [18], among other possibilities, such as a regulatory effect of ATP on P2Y2/P2Y6 receptor function. Distinguishing among the various possibilities will require further analysis of the functional interaction among the different P2 receptors expressed in the ovarian theca.

Data presented in the present work are the first evidence that UTP-sensitive P2Y receptors are expressed and functional in theca cells. Although extensive studies are necessarily to establish with detail the main physiological activities, experimental data suggested these receptors have a role in p44/p42 MAPK phosphorylation, proliferation increase, and cross talk with LH-activated pathways. These observations raise the possibility that the purinergic signaling system represents an important physiological regulator of theca cells.

## Conclusion

In summary, it was shown here that TIC express functional P2Y2 and P2Y6 receptors, which, when stimulated, induce a Ca^2+^-dependent proliferative response mediated through PKC activation and phosphorylation of the p42 and p44 MAPK proteins. P2Y receptor stimulation also regulates hCG-dependent CREB phosphorylation, suggesting interactions between functional pathways. Molecular components of purinergic transmission systems represent new molecular targets that must be characterized in the context of ovarian pathophysiology.

## List of abbreviations

P2Y: Purinergic G protein-coupled receptors; MAPK: Mitogen-activated protein kinases; PPADS: pyridoxal phosphate-6-azo (benzene-2,4-disulfonic acid) tetrasodium salt; BAPTA: 1,2-bis(o-aminophenoxy)ethane-N, N, N', N'-tetraacetic acid; CREB: cAMP response element binding protein; TIC: Theca interstitial cells; PMA: Phorbol 12-myristate 13-acetate.

## Competing interests

The authors declare that they have no competing interests.

## Authors' contributions

FGVC and ROA conceived and designed the study, carried out experiments, performed the data analysis, and drafted the manuscript; EPZD and GE performed experiments and participated in the analysis of data. All authors read and approved the final manuscript.
